# Cytokeratin 20 (CK20) and apomucin 1 (MUC1) expression in ampullary carcinoma: Correlation with tumor progression and prognosis

**DOI:** 10.1186/1746-1596-5-75

**Published:** 2010-11-25

**Authors:** Yasunari Kawabata, Tsuneo Tanaka, Takashi Nishisaka, Touko Inao, Takeshi Nishi, Seiji Yano

**Affiliations:** 1 Department of Digestive and General Surgery, Shimane University Faculty of Medicine,89-1 Enyacho, Izumo, Shimane 693-8501, Japan; 2 Department of Pathology, Hiroshima Prefectural Hospital, 1-5-54 Ujinakanda, Minamiku, Hiroshima 734-8530, Japan

## Abstract

**Background:**

We assessed the expression of cytokeratin (CK) and apomucin (MUC) in ampullary carcinoma (AC) to develop a system for the classification of ACs on the basis of their clinical significance.

**Method:**

We studied the expressions of CK7, CK20, MUC1, MUC2, MUC5AC, and MUC6 in 43 patients with ACs. Clinical data were obtained retrospectively by examining surgically resected ACs of the patients.

**Results:**

We classified the cases into 3 groups: tumors expressing CK20 and lacking MUC1 (intestinal type [I-type], 26%), tumors expressing MUC1 and lacking CK20 (pancreatobiliary type [PB-type], 35%), and those expressing or lacking both CK20 and MUC1 (other type [O-type], 39%). Eight (73%) of 11 I-type carcinomas, 3 (20%) of 15 PB-type carcinomas, and 4 (24%) of 17 O-type carcinomas were classified as pT1. The number of I-type carcinomas in the early tumor stages was significantly higher than the number of PB- and O-type carcinomas (p = 0.014 and p = 0.018, respectively). The 5-year survival rates for pT1, pT2, and pT3 tumors were 76%, 33%, and 22%, respectively (p < 0.001). Rates of MUC5AC and MUC6 coexpression for I-type, PB-type, and O-type tumors were 18%, 13%, and 53%, respectively. There was a significant correlation between MUC5AC and MUC6 coexpression and O-type characteristics (p = 0.031). The five-year survival rates for O-type ACs with and without MUC5AC and MUC6 coexpression were 71% and 17%, respectively (p = 0.048).

**Conclusions:**

The immunohistochemical subtypes based on CK and MUC expression correlated with tumor progression. Gastric MUC5AC and MUC6 coexpression correlated with better prognosis for O-type ACs.

## Background

Ampullary carcinomas (ACs), although uncommon, have a better prognosis than other periampullary tumors such as pancreatic and bile duct carcinomas.

The ampulla of Vater consists of 4 minor anatomic regions: the ampulloduodenum (Ad), the ampullopancreatobiliary common duct (Ac), the ampullopancreatic duct (Ap), and the ampullobiliary duct (Ab)[1,2]. The ampulla is formed by the union of 2 distinct types of mucosa. The Ad is covered by intestinal mucosa, while the other parts of the ampulla of Vater (the Ap, Ab, and Ac) are lined with pancreatobiliary-type ductal mucosa[2,3]. Therefore, ACs may arise from the intestinal-type mucosa as well as from the pancreatobiliary-type mucosa; this may explain the broad histomorphologic spectrum of these tumors[1]. Tumor progression and prognosis are affected by the primary AC tumor sites [1,3,4].

Kimura et al. classified ACs into 2 histological subtypes: intestinal and pancreatobiliary[3]. Albores-Saavedra et al. further defined the characteristics of these 2 types and also described unusual types such as signet-ring cell carcinoma and undifferentiated carcinoma[4].

While histopathological typing is a useful method for classifying ACs, some cases cannot be easily classified by using histomorphology[1]. Determination of the cytokeratin (CK) and apomucin (MUC) immunophenotypes of an AC can facilitate identification of the primary tumor site[1,2,5-7]. Most pancreatobiliary adenocarcinomas express CK7 and low levels of CK20[6,8,9]. Among ACs, the pancreatobiliary type expresses CK7 but does not express CK20, while the intestinal type expresses CK20 but does not express CK7[5].

The pancreatobiliary type of ACs usually express MUC1 but do not express MUC2[6,7,9,10]. Most intestinal-type ACs express MUC2[1,2,5,6].

In the present study, we analyzed the spectrum of CK and MUC expression in 43 patients with ACs. We then evaluated the immunohistochemical subtypes of ACs by analyzing the expressions of CK7, CK20, MUC1, MUC2, MUC5AC, and MUC6 in these tumors. Further, we assessed the correlations between the histomorphological findings and the defined immunohistochemical subtypes and evaluated the clinical significance of these immunohistochemical AC subtypes; the classification of ACs on the basis of their immunohistochemical characteristics may be useful to predict the clinical outcome.

## Materials and methods

Clinical data were obtained retrospectively from ACs that were surgically resected from 43 patients (22 men and 21 women) with an average age of 66.4 years (range, 44-82 years). All resected specimens had been obtained between 1983 and 2007 and were maintained at the Department of Digestive and General Surgery, Faculty of Medicine, Shimane University. All but 5 patients underwent pancreatoduodenectomy. The other 5 underwent pancreas-sparing duodenectomy. All tumors included in this study histologically showed surgically negative margins.

The study was approved by the hospital's ethics committee. Informed consent was obtained from all patients for the subsequent use of resected tissues.

**Figure 1:**
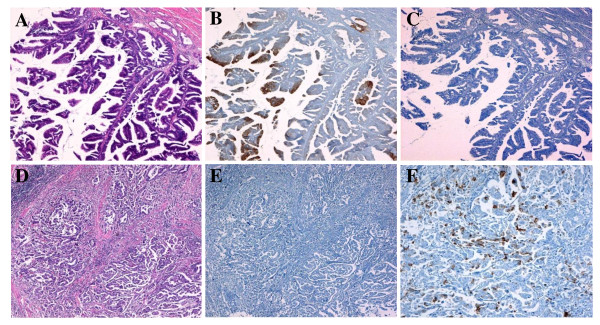
**Expression of cytokeratin and apomucin for histological classification**. Intestinal-type carcinomas are composed of well-formed tubular to elongated glands and complex cribriform areas indistinguishable from colorectal adenocarcinomas (A), and they are immunohistochemically stained by the CK20 antibody (B) but show no reaction to the MUC1 antibody (C). Pancreatobiliary-type carcinomas mostly consist of simple or branching glands surrounded by strikingly desmoplastic stroma (D) negative for CK20 (E) and positive for MUC1 antibody (F) (A, D: hematoxylin and eosin stain, ×20; B, C, E: ×20;F ×50).

Histopathological examinations were performed according to the guidelines of the Japanese Society of Biliary Surgery[11]. The Tumor, Node, Metastasis (TNM) Staging System put forth by the International Union Against Cancer was used for tumor classification[12]. All tumors were classified histologically according to the criteria published by Albores-Saavedra et al[4]. Intestinal-type carcinomas are composed of well-formed tubular to elongated glands, complex cribriform areas, and solid nests indistinguishable from those found in colorectal adenocarcinoma, whereas pancreatobiliary-type carcinomas mostly consist of simple or branching glands and small solid nests of cells surrounded by a strikingly desmoplastic stroma (Figure 1). Mixed-pattern tumors were classified into the intestinal- or pancreatobiliary-type groups on the basis of their predominant component. Carcinomas of the unusual types included undifferentiated, mucinous, signet-ring cell, and solid carcinomas.

### Histological and Immunohistochemical Staining

All specimens were fixed in 4% buffered formaldehyde and embedded in paraffin. The samples were sectioned (section thickness, 3 μm) and stained with hematoxylin and eosin. Subsequently, all tissue samples, including carcinoma tissues, were stained immunohistochemically with the following antibody panel: monoclonal antibodies to cytokeratin 7 (CK7, OV-TL12/30, dilution 1:100; Dako Cytomation, Carpinteria, CA), cytokeratin 20 (CK20, Ks20.8, dilution 1:50; Dako Cytomation), apomucin MUC1 (clone Ma695, 1:100; Novocastra, Mount Waverley, Australia), apomucin MUC2 (clone Ccp58, 1:100; Novocastra), apomucin MUC5AC (clone CLH2, 1:150; Novocastra), and apomucin MUC6 (clone CLH5, 1:150; Novocastra).

Immunohistochemical staining was performed on an immunostainer (Benchmark XT; Ventana Medical System, Tucson, AZ) with the use of an amplification kit (Ventana). Antibody detection was performed by adding biotinylated secondary antibodies, avidin-biotin complex, and 3,3'-diaminobenzidine.

The cytoplasmic and membranous immunoreactivities of CK7, CK20, MUC1, MUC2, MUC5AC, and MUC6 were assessed. Only those samples showing greater than 10% tumor-cell positivity were regarded as positive.

### Statistical Analysis

Survival curves were calculated by the Kaplan-Meier method and compared with the results of the log-rank test. Agreement between the histological and immunohistochemical classifications was evaluated using the κ-coefficient. A two-tailed Fisher's exact test or χ^2 ^test was used to compare the immunohistochemical classification and clinicopathological parameters, as appropriate. Probability (p) values of < 0.05 obtained by the two-tailed test were regarded as statistically significant.

The JMP software program (ver. 5.0.1; SAS Institute Inc, Cary, NC) was used for all statistical analyses.

## Results

### Patient Characteristics and Histological Classification

Fifteen (35%) of the 43 tumors were pT1, 11 (25%) were pT2, and 17 (40%) were pT3. Twenty-two (51%) and 21 (49%) of the 43 tumors were negative and positive for lymph node involvement, respectively.

In the assessment according to the histological criteria proposed by Albores-Saavedra et al.,[4] we found 16 (37%) tumors to be intestinal-type carcinomas, 18 (42%) to be pancreatobiliary-type carcinomas, and 9 (21%) to be unusual-type carcinomas (Table 1).

**Table 1:** Clinicopathological Data of 43 Ampullary Carcinomas

Median age, years (range)	66.4 (44-82)	
Gender		
		
Male	22	51.0%
Female	21	49.0%
Histological classification*		
		
Intestinal type	16	37.0%
Pancreatobiliary type	18	42.0%
Unusual type	9	21.0%
T stage		
		
I	15	35.0%
II	11	25.5%
III	17	39.5%
IV	0	0.0%
Nodal metastasis		
Negative	21	49.0%
Positive	22	51.0%

*according to reference [4].

### Correlations between Histological Classification and Immunohistochemical Parameters

The histological classification[4] indicated that CK20 had high sensitivity (100%) for intestinal-type carcinoma and that MUC1 had high sensitivity (94%) for pancreatobiliary-type carcinoma (Table 2, Figure 1), and both correlations were significant (p < 0.001 and p < 0.001, respectively).

**Table 2:** Immunohistochemical Expression by Histological Classification

Positive cases (%)				
	Intestinal type	Pancreatobiliary type	Unusual type	P*
	(N = 16)	(N = 18)	(N = 9)	
CK7	13 (81)	17 (94)	9 (100)	(p = 0.178)
CK20	16 (100)	4 (22)	6 (67)	(p < 0.001)
MUC1	6 (38)	17 (94)	7 (78)	(p < 0.001)
MUC2	4 (25)	2 (11)	2 (22)	(p = 0.540)
MUC5AC	6 (38)	8 (44)	5 (56)	(p = 0.683)
MUC6	7 (44)	5 (28)	5 (56)	(p = 0.341)

* χ^2 ^test. The percentage represents sensitivity

### Immunohistochemical Classification of Tumors

We tried to further classify ACs into 3 subtypes on the basis of the expression of CK20 and MUC1 in the intestinal mucosa and the pancreatobiliary mucosa: tumors expressing CK20 and lacking MUC1 were defined as intestinal type (I-type); tumors expressing MUC1 and lacking CK20 were defined as pancreatobiliary type (PB-type); and carcinomas expressing or lacking both CK20 and MUC1 were defined as other type (O-type). Eleven (26%) of the 43 tumors were I-type, 15 (35%) were PB-type, and 17 (39%) were O-type.

### Correlation Analysis of Histological and Immunohistochemical Classifications

Ten of the 16 carcinomas of the histological intestinal type were of the immunohistochemical I-type, but none was of the immunohistochemical PB-type. Thirteen of the 18 carcinomas of the histological pancreatobiliary type were of the immunohistochemical PB-type, while none was of the immunohistochemical I-type (Table 3). The sensitivity of the marker for the coexpression of CK20 and MUC1 for the histological intestinal type, pancreatobiliary type, and unusual type of tumors were 63%, 72%, and 67%, respectively, and the specificity for these types were 96%, 92%, and 68%, respectively (Table 4). Further, the immunohistochemical subtypes defined in our study correlated well with the conventional histological classification (κ-coefficient = 0.518; p < 0.001).

**Table 3:** Correlation between Immunohistochemical and Histological Classifications

	Immunohistochemical classification		
	
	I-type	PB-type	O-type
Histological classification			
Intestinal type	10	0	6
Pancreatobiliary type	0	13	5
Unusual type	1	2	6

I-type: intestinal type; PB-type: pancreatobiliary type; O-type: other type

**Table 4:** Sensitivity, Specificity, Positive Predictive Value, and Negative Predictive Value of Combined Expression of CK20 and MUC1 According to the Histological Type

Histological type	Maker	Sensitivity	Specificity	PPV	NPV
Intestinal type					
	CK20+MUC1	0.625	0.963	0.909	0.813
Pancreatobiliary type					
	CK20+MUC1	0.722	0.920	0.867	0.821
Unusual type					
	CK20+MUC1	0.666	0.676	0.353	0.885

PPV, positive predictive value; NPV, negative predictive value

### Correlations between Immunohistochemical Classification and Tumor Parameters

Eight (73%) of the 11 I-type tumors, 3 (20%) of the 15 PB-type tumors, and 4 (24%) of the 17 O-type tumors were classified as pT1 according to the immunohistochemical classification system. Lymph node metastasis was observed in 3 (27%) of the 11 I-type ACs, 9 (60%) of the 15 PB-type ACs, and 9 (53%) of the 17 O-type ACs (Table 5). Thus, the immunohistochemical I-type had significantly better pT stage than the immunohistochemical PB-type and O-type tumors (Fisher's exact test, p = 0.014 and p = 0.018, respectively) (Table 6). However, there were no significant differences in the frequency of lymph node metastasis among the immunohistochemical subtypes. (Fisher's exact test, p = 0.130 and p = 0.253, respectively)

**Table 5:** Correlations of Tumor Parameters with Both Histological and Immunohistochemical Classifications

	Histological type (No. of cases)			Immunohistochemical type (No. of cases)		
		
	I-type	PB-type	U-type	I-type	PB-type	O-type
	(N16)	(N = 18)	(N = 9)	(N = 11)	(N = 15)	(N = 17)
T-stage						
I	10	4	1	8	3	4
II	4	5	2	0	3	8
III	2	9	6	3	9	5
IV	0	0	0	0	0	0
Nodal metastasis						
Negative	13	7	2	8	6	8
Positive	3	11	7	3	9	9

I-type, intestinal type; PB-type, pancreatobiliary type; U-type, unusual type; O-type, other type

**Table 6:** Relationships between Tumor Groups and Tumor Parameters

	Two-Tailed P*		
	
Tumor parameters	I-type vs. PB-type type	PB-type vs. O-type	I-type vs. O-type
Histological classification			
T1 vs. T2/T3	0.017	0.483	0.013
N0 vs. N1	0.012	0.386	0.003
Immunohistochemical classification			
T1 vs. T2/T3	0.014	0.809	0.018
N0 vs. N1	0.130	0.734	0.253

* Fisher's exact test.

Other type implies histological unusual type.

### Survival Analysis

**Figure 2:**
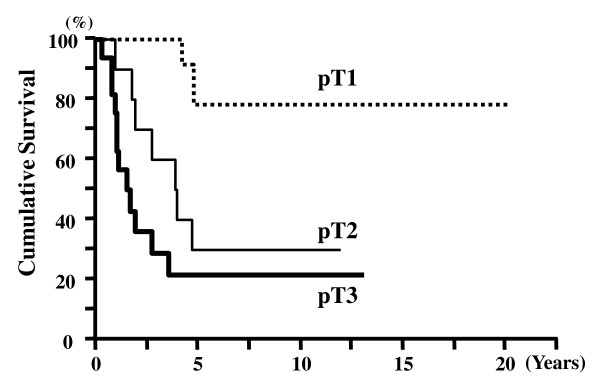
**Cumulative survival of patients according to pathological tumor stage (pT)**. Patients with early-stage tumors (pT1) showed significantly better survival than those with advanced-stage tumors (pT2 and pT3) (p < 0.001).

**Figure 3:**
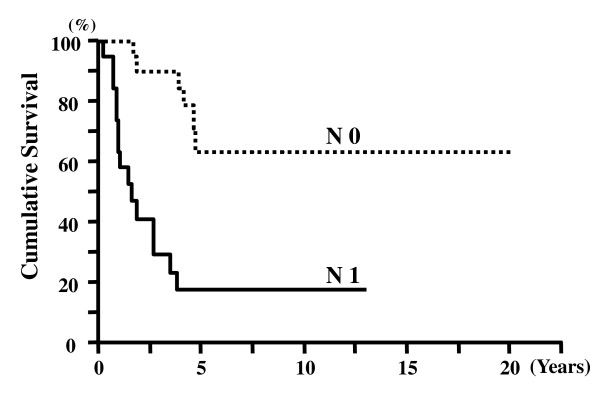
**Cumulative survival of patients with lymph node metastasis (N)**. Node-positive (N1) patients showed significantly poorer survival than node-negative (N0) patients (p < 0.001).

**Figure 4:**
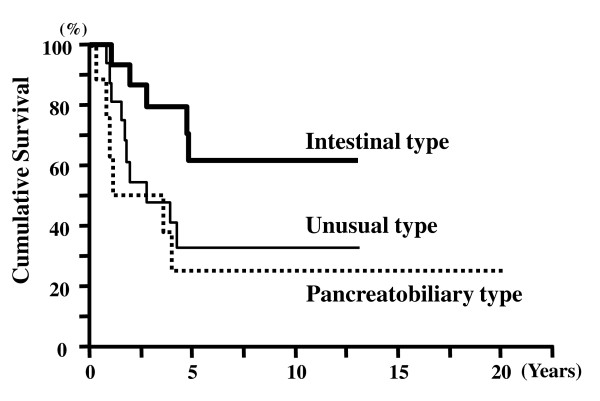
**Cumulative survival of patients with ACs of different histological types: intestinal-type, pancreatobiliary-type, and unusual-type**. Patients with intestinal-type tumors showed significantly better survival than those with the pancreatobiliary- and unusual-type tumors (p = 0.031).

**Figure 5:**
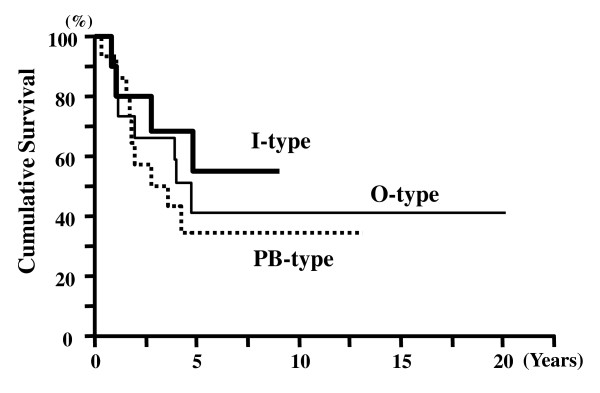
**Cumulative survival of patients based on immunohistochemical classification (I-type, PB-type, and O-type)**. There were no differences among the 3 immunohistochemical subtypes in terms of cumulative survival of patients with AC (p = 0.56).

The survival time of the 43 patients was 4.23 ± 0.64 years (mean ± SEM, Kaplan-Meier). The 5-year survival rate for all AC cases was 41.2%. The 5-year survival rates for pT1, pT2, and pT3 cases were 76%, 33%, and 22%, respectively (Figure 2). The 5-year survival rates for node-negative and node-positive cases were 61% and 19%, respectively (Figure 3). There was a significant difference in the cumulative survival between pT and N cases (p < 0.001 and p < 0.001, respectively). The 5-year survival rates for the histological intestinal, pancreatobiliary, and unusual types were 62%, 33%, and 25%, respectively (p = 0.034) (Figure 4). However, the 5-year survival rates for the immunohistochemical I-type, PB-type, and O-type were 55%, 35%, and 41%, respectively; therefore, we concluded that there was no significant correlation between survival and immunohistochemical subtypes (p = 0.560) (Figure 5).

### Relationships between MUC5AC and MUC6 Expression and Immunohistochemical Subtypes

**Figure 6:**
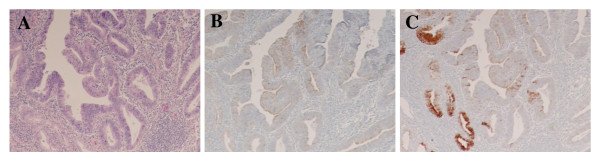
**Hematoxylin and eosin staining of immunohistochemical other-type tumors (A)**. Positive expression of MUC5AC antibody (B) and MUC6 antibody (C) in immunohistochemical other-type tumors (A: ×20; B, C: ×20).

The rate of coexpression of MUC5AC and MUC6 in tumors of the immunohistochemical I-type, PB-type, and O-type were 18%, 13%, and 53%, respectively. Significant correlations were noted between MUC5AC and MUC6 coexpression and immunohistochemical subtypes (p = 0.031) (Figure 6) (Table 7). For the immunohistochemical O-type, the 5-year survival rates of patients with tumors coexpressing MUC5AC and MUC6 and with those lacking both antigens were 71% and 17%, respectively.

**Table 7:** Correlation of MUC5AC and MUC6 Coexpression with Immunohistochemical Classification

	I-type	PB-type	O-type	P*
	(N = 11) (%)	(N = 15) (%)	(N = 17) (%)	
MUA5AC and MUC6	18	13	53	p = 0.031

* χ^2 ^test

One (11%) of the 9 tumors positive for MUC5AC and MUC6 coexpression and 4 (50%) of the 8 tumors negative for MUC5AC and MUC6 coexpression were classified into stage pT3 (Table 8).

**Table 8:** Correlation of Tumor Parameters and MUC5AC and MUC6 Coexpression with Immunohistochemical O-type

	O-type(No. of cases)	
	
	MUC5AC +/MUC6 +	MUC5AC -/MUC6 -
	(N = 9)	(N = 8)
T-stage		
I	3	1
II	5	3
III	1	4
IV	0	0
Nodal metastasis		
Negative	5	3
Positive	4	5

**Figure 7:**
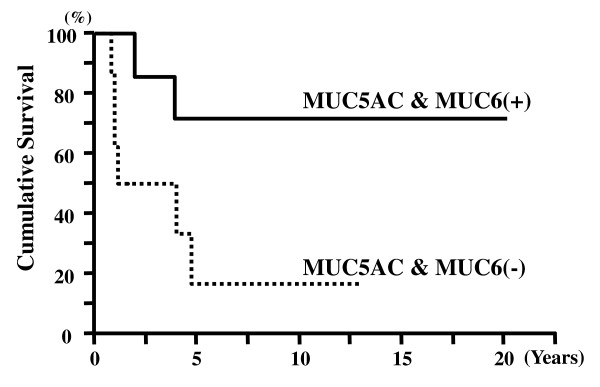
**Cumulative survival of patients with immunohistochemical other-type tumors**. Patients with tumors showing coexpression of MUC5AC and MUC6 showed significantly better survival than those with tumors negative for expression of both MUC5AC and MUC6 (p = 0.048).

Among the patients with immunohistochemical O-type tumors, those with tumors coexpressing MUC5AC and MUC6 had significantly longer cumulative survival than those with tumors that did not show this coexpression (p = 0.048) (Figure 7).

## Discussion

Albores-Saavedra et al. defined 3 types of ACs--the intestinal type, the pancreatobiliary type, and the unusual type[4].

In the present study on ACs, we histologically classified these tumors into these 3 types, and 37% were of the intestinal type, 42% were of the pancreatobiliary type, and 21% were of the unusual type[4]. According to the histological criteria, Zhou et al. reported the rates of the histological intestinal, pancreatobiliary, and unusual types to be 27%, 44%, and 29%, respectively[5]. According to their histological criteria, the unusual type included mucinous, signet-ring cell, solid, or undifferentiated carcinomas[4,5]. However, Kimura et al. simply classified ACs into 2 types, intestinal and pancreatobiliary[3].

Because these systems of histological classification use different sets of criteria,[3,4] we tried to create a simple AC classification system based on immunohistochemical staining of CKs and MUCs. Goldstein et al. reported positive CK7 expression in 100% and positive CK20 expression in 43% of ACs, but it was difficult to distinguish between pancreatic carcinomas and ACs by the coordinate staining patterns of CK7 and CK20[9]. Very few reports have examined the expression patterns of CK and MUC in the AC subtypes[1,5,6].

In a previous study, the CK7+/CK20-/MUC2- pattern in the histological intestinal-type carcinoma and the CK7-/CK20+/MUC2+ pattern in the histological pancreatobiliary-type carcinoma indicated that these different types of ACs had developed from 2 different types of mucosa in the ampulla of Vater[1]. Chu et al. reported positive expression of CK7, CK20, and MUC2 in the histological intestinal-type and positive expression of CK7 and MUC1 in the histological pancreatobiliary-type and that ACs of pancreatobiliary origin showed immunophenotypes similar to that of pancreatic ductal carcinoma[6]. Zhou et al. were the first to show agreement between the histological classification and the immunohistochemical characterization based on cytokeratins[5]. However, their immunohistochemical classification did not correlate with tumor progression and prognosis.

Although most other studies have described immunohistochemical classification systems based on the expression of either MUC[2] or CK[5], we analyzed the expression of both in ACs.

In the present study, significant differences were noted in the expression levels of the histological intestinal type and the histological pancreatobiliary type, with the sensitivity being 100% for CK20 and 94% for MUC1 expression, respectively. These results indicate that the CK20+/MUC1- pattern fully corresponds to the immunohistochemical I-type and that the CK20-/MUC1+ pattern fully corresponds to the immunohistochemical PB-type.

With regard to immunohistochemical classification systems, Zhou et al classified ACs on the basis of the combined expression of CK7 and CK20, while Chu et al classified ACs on the basis of the combined expression of CDX2, CK17, MUC1, and MUC2.

Little is known, however, about the combined expression of CK20 and MUC1 in ACs. The possibility of identifying the primary AC site is increased when the combined expression of CK and MUC, rather the expression of either one of them, is taken into account.

We found that the classification of the immunohistochemical subtypes based on the expression of both CK20 and MUC1 correlated well with histological typing (κ-coefficient = 0.5184). Using this immunohistochemical classification system based on the coordinated expression of CK20 and MUC1, we were able to determined that 2 of 9 tumors classified as the histological unusual type expressed the pancreatobiliary pattern in immunohistochemical analysis, while 1 of these 9 tumors expressed the intestinal pattern.

Previous studies have shown that the prognosis of AC patients depends on the pT stage, nodal metastasis, and histological type[1,4,13-17]. Similarly, our results indicated that the pT stage, nodal metastasis, and histological subtype correlated significantly with cumulative survival. According to our histological classification method, a large number of intestinal type ACs were at stage pT1 (60%) and node-negative (81%). These results indicate that progression of the intestinal-type tumor is slower and the risks of nodal metastasis lower than those in the case of the pancreatobiliary- and unusual-type tumors.

In our immunohistochemical classification, the pT stage correlated significantly with the immunohistochemical subtypes. Although the number of immunohistochemical I-type tumors in the early pT stages was significantly greater than the number of immunohistochemical PB-type and O-type tumors in the same stages, there were no significant differences in the cumulative survival. This result may be attributable to the lack of differences in the nodal metastasis risks associated with the immunohistochemical I-type and PB-type. This may indicate that the risks of nodal metastasis are similar among the intestinal, pancreatobiliary, and other types. Therefore, pancreatoduodenectomy with lymph node dissection should be performed for adequate surgical resection in AC[18]. If function-preserving surgery has been selected for AC, the histological classification system is currently more useful than our immunohistochemical classification method.

Gürbüz et al. reported that MUC5AC- and MUC6-positive expression patterns were regarded as representing gastric differentiation and that negative expression of both indicated the intestinal type of AC[7]. Zhou et al. showed that gastric MUC5AC expression correlated well with PB-type carcinomas[5]. Interestingly, we found that the frequency of the coexpression of gastric MUC5AC and MUC6 in the immunohistochemical O-type was significantly higher than those in the immunohistochemical I-type or PB-type. These results provide evidence that both the immunohistochemical I-type and immunohistochemical PB-type have low-grade expression of gastric mucins. Expression of these gastric MUCs in the immunohistochemical O-type indicates that both gastric foveolar and pyloric gland metaplasia occur in the immunohistochemical O-type[19-21]. In addition, patients with tumors of the immunohistochemical O-type and MUC5AC and MUC6 coexpression had a significantly longer survival than those with tumors that did not show this coexpression. Among the tumors of the immunohistochemical O-type, those negative for the coexpression were at a more advanced pT stage than those positive for the coexpression. The prognosis of tumors of the immunohistochemical O-type that were positive or negative for the coexpression may depend on the tumor stage. Thus, gastric differentiation of the immunohistochemical O-type is associated with good prognosis. Interestingly, our observations of the histological unusual type were similar.

In summary, we studied the expression of CK7, CK20, MUC1, MUC2, MUC5AC, and MUC6 in ACs. On the basis of the histological classification of ACs, we found that CK20 had a high sensitivity for the histological intestinal type and MUC1 had high sensitivity for the histological pancreatobiliary type carcinoma. On the basis of the observed differences in the expression patterns of both CK and MUC, we defined immunohistochemical subtypes. These immunohistochemical subtypes correlated well with the conventional histomorphological classification but did not correlate with prognosis. However, the coexpression of gastric MUC5AC and MUC6 correlates with the prognosis of patients with the immunohistochemical O-type of AC.

## Competing interests

The authors declare that they have no competing interests.

## Authors' contributions

YK participated in the design of the study, histological diagnoses, data processing, and drafting of the manuscript. TT participated in the design of the study and reviewed the manuscript. TN established histological diagnoses and reviewed the manuscript. TI, TN, and SY participated in the design of the study, established histological diagnoses, and reviewed the manuscript. All authors read and approved the final manuscript.
